# Navigating the Dry Tap Conundrum: A Successful Spinal Anesthesia for a Cesarean Section

**DOI:** 10.7759/cureus.46726

**Published:** 2023-10-09

**Authors:** Saleh A Ba-shammakh, Ahmad Al-samnah, Mohammad K Zidan, Haitham M Abdallah

**Affiliations:** 1 Department of General Surgery, Princess Basma Teaching Hospital, Irbid, JOR; 2 Department of Anesthesiology and Reanimation, The Islamic Hospital, Amman, JOR

**Keywords:** intrathecal, anesthesiology, cesarean section, dry tap, spinal anesthesia

## Abstract

A "dry tap" in spinal anesthesia is characterized by the lack of cerebrospinal fluid (CSF) after needle insertion and poses unique challenges for anesthesiologists. We present an uncommon case of a 30-year-old female undergoing a cesarean section who experienced this situation. Despite the absence of CSF after several attempts, the patient's sensory alterations post-anesthesia administration confirmed intrathecal placement. This successful administration of spinal anesthesia in the face of a dry tap emphasizes the value of clinical observation and adaptability, offering an innovative perspective on addressing such rare occurrences.

## Introduction

Spinal anesthesia, also known as subarachnoid block, is a prevalent form of neuraxial anesthesia frequently used for surgical procedures below the umbilicus or those involving the lower extremities [1]. Experienced anesthesiologists can typically recognize the sensation of the needle advancing through high-resistance ligaments, feeling a subtle "pop" or "give way" as it enters the subarachnoid space. A successful block is confirmed by the free flow of clear cerebrospinal fluid (CSF) from the needle's hub upon needle withdrawal [1]. However, anesthesiologists face substantial challenges when no CSF appears after needle placement, a situation known as a "dry tap" [2]. Although dry taps are relatively rare, they complicate the administration of subarachnoid blocks. Published accounts of successfully executed blocks post-dry tap without complications are scarce. In this report, we discuss a case of a successful subarachnoid block following a dry tap in a 30-year-old female patient undergoing a cesarean section.

## Case presentation

**Figure 1:**
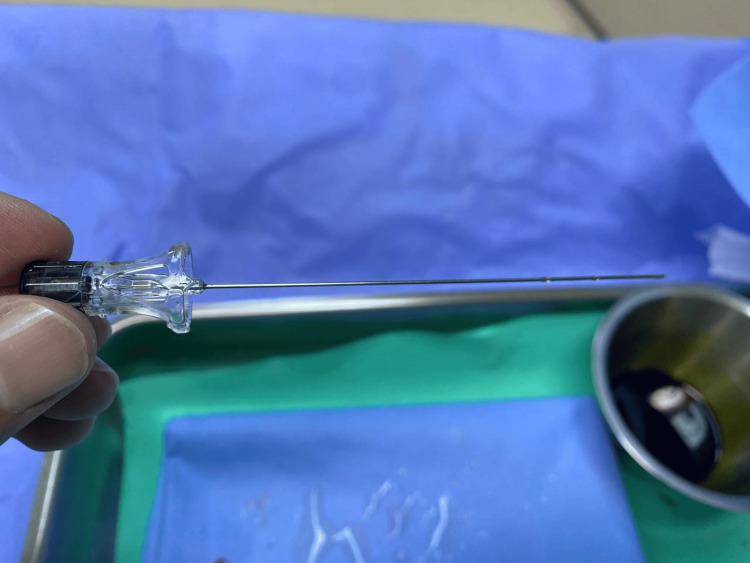
25-gauge Sprotte needle None-cutting

A 30-year-old female - gravida 3, para 2 (G3P2) - with no history of medical or surgical complications and two prior normal vaginal deliveries was scheduled for a cesarean section due to breech presentation. The patient had never previously been exposed to spinal or epidural anesthesia. Upon presentation, her vital signs were within normal limits, and she received 500 ml of intravenous normal saline for preoperative hydration. Skin preparation was performed using an aseptic technique with povidone-iodine, and local anesthesia was administered with 3 cc of 2% lidocaine. Initial attempts at spinal anesthesia were made while the patient was in a sitting position; she was later transitioned to a left lateral position. The L4-L5 interspace was initially targeted using a 25-gauge Sprotte needle (Figure 1). Despite the loss of resistance observed in two trials, indicative of successful penetration of the ligamentum flavum, no CSF backflow was observed.

**Figure 2:**
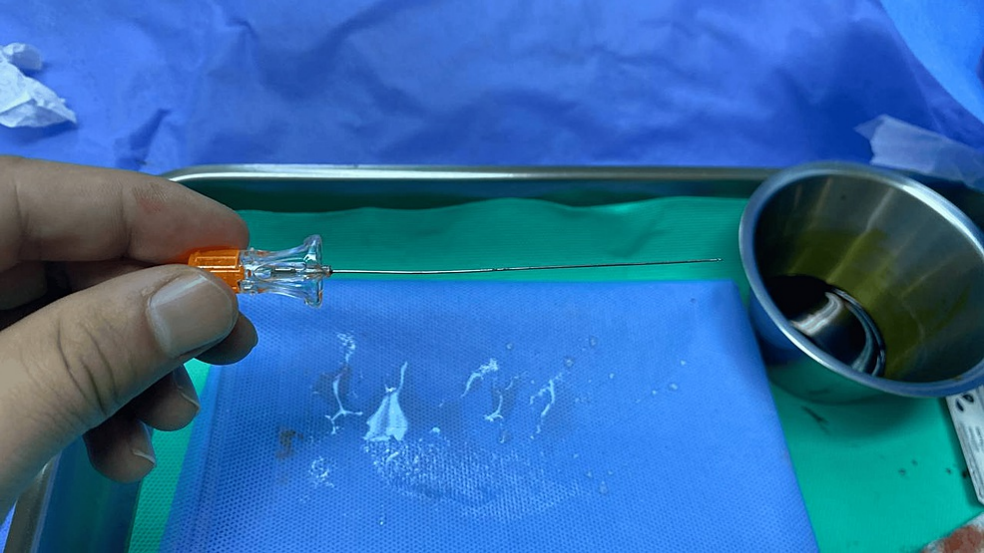
22-gauge Quincke needle Cutting

Subsequent procedures were then executed thrice at the L3-L4 interspace using a 22-gauge Quincke needle (Figure 2). Although a loss of resistance was noted in each trial, CSF backflow was still not observed. Throughout these procedures, the needle successfully penetrated the ligamentum flavum, entering the subarachnoid space. Both needle malfunction and patient anatomical malformation were excluded as potential contributing factors.

In the final attempt, a 25-gauge Sprotte needle was introduced at the L4-L5 interspace where a loss of resistance was achieved. However, due to the absence of CSF backflow, a test dose of 1 cc of bupivacaine 0.5% was cautiously administered. The patient soon reported experiencing paresthesia and warmth in her lower legs. An additional 2 cc of bupivacaine 0.5% was administered, rendering a total dose of 3 cc. Subsequently, a complete motor block was achieved, with the patient experiencing sensory blockade up to the lower border of the scapula at T7.

The cesarean section was successfully performed, and both the mother and newborn were stable postoperatively. There was no requirement for additional sedation or analgesics.

## Discussion

The phenomenon of a "dry tap" during spinal anesthesia represents a conundrum, raising concerns and requiring adaptation on the part of anesthesiologists. Although rare, dry taps following needle placement into the subarachnoid space demand astute clinical reasoning and immediate action [2-3]. These dry taps can stem from various causes, such as obstructions or improper positioning of the needle, as well as anatomical and physiological conditions like previous spine surgeries, reduced CSF pressure, or the closure of the subarachnoid space due to the arachnoid layer pressing against the pia mater [2].

Lack of CSF may result from conditions like decreased CSF pressure or dehydration, leading to the compression of the arachnoid membrane and enlargement of the subdural space [3]. Addressing these challenges requires reliable methods for verifying needle positioning accurately. Tsui et al. introduced a noteworthy technique that uses an electrically insulated needle to provide real-time confirmation of needle placement within the intrathecal or subdural region, regardless of CSF presence [4-5]. For dry tap cases, it is advisable to consider real-time imaging modalities such as fluoroscopic or CT scans for diagnostic or therapeutic purposes [6]. Pre-procedural ultrasonography for assessing vertebral anatomy reportedly enhances the success rate of spinal blocks, even in dry tap cases [7-8].

In our case, successful spinal block execution was achieved despite CSF absence after the patient reported sensory changes -paresthesia and warmth- following a trial dose of bupivacaine 0.5%. These sensory alterations indicated intrathecal placement, guiding the decision to administer the complete anesthetic dose, in line with previous accounts of successful spinal anesthesia despite dry taps [2,9]. It is crucial to acknowledge that the absence of CSF confirmation for needle placement carries certain risks. Complications like spinal subdural hematoma or subdural anesthesia leading to high spinal blocks have been reported in cases where multiple attempts to locate the subarachnoid space resulted in dry taps [10]. In high-risk situations, such as suspected epidural abscesses, it would be prudent to avoid local anesthetic injections unless other diagnostic modalities confirm safe needle placement [3].

Navigating a dry tap during spinal anesthesia is challenging. Still, advancements in techniques and a deeper understanding of its causes can guide anesthesiologists in making informed clinical decisions. The successful spinal block in this case demonstrates that careful clinical observation and innovative techniques can ensure positive outcomes in such scenarios.

## Conclusions

The rarity and intricacy of a dry tap in spinal anesthesia necessitate rigorous clinical judgment and adaptability from practitioners. While traditional methods prioritize CSF backflow as a marker for a successful subarachnoid block, this case highlights the possibility of success even in the absence of CSF. Patient-reported sensory changes provided alternative confirmation, leading to the successful administration of spinal anesthesia. This case not only sheds light on the multifaceted nature of a dry tap but also underscores the importance of a nuanced approach that integrates classical teachings with innovative strategies, thereby ensuring patient safety and the effective delivery of anesthesia.
